# Barriers to seeking and delivery of essential health services in nine provinces of Afghanistan during the COVID-19 pandemic: community health workers’ perspective

**DOI:** 10.1186/s12913-025-12841-3

**Published:** 2025-05-07

**Authors:** Freshta Amiry, Narges Neyazi, Ali Mirzazadeh, Abdul Ghani Ibrahimi, Deena AlAsfoor, Jamshed Ali Tanoli

**Affiliations:** 1 MPH International, Nuffield Centre for International Health and Development, School of Medicine, University of Leeds, Leeds, UK; 2 Health System Development Department, World Health Organization, Kabul, Afghanistan; 3 Department of Epidemiology and Biostatistics, Institute for Global Health Sciences, University of California, San Francisco, CA USA; 4 Department of Universal Health Coverage, Eastern Mediterranean Regional Office, World Health Organization, Cairo, Egypt; 5 Country Representative, World Health Organization, Kabul, Afghanistan

**Keywords:** COVID-19 pandemic, Community health workers, Essential health services, Barriers, Delivery, Rural health care

## Abstract

**Introduction:**

Community health workers (CHWs) played a vital role in providing diverse essential health services to their communities during the pandemic. Using CHWs perspective, this study investigates barriers to seeking and delivery of essential health services in the nine Afghan communities during the COVID-19 pandemic.

**Method:**

In this cross-sectional study, 107 primary health care clinics from 9 provinces were selected, in which around 45% of the total country population reside. We used the validated questionnaire “Community Needs, Perceptions and Demand, Community Assessment Tool” which was contextualized by WHO headquarters and the WHO Afghanistan office. Data was exported into Excel, cleaned, and then exported into and analyzed using STATA version 17.

**Result:**

Most CHWs were men (80.9%), from rural areas (87.2%), and volunteers (58.1%). About 66.3% reported that lack of information about available services was a main barrier. Other reported barriers were a lack of transportation to health facilities (47.2%), their home were too far from health facilities (40.9%), and a perceived lack of medicines at facilities (23.6%). More than half of CHWs reported that they received some training on how COVID-19 spreads (67.2%), COVID-19 vaccine (65.4%), and how to use a mask properly while working (56.3%), while 27.2% said that they had not enough mask available to use.

**Conclusion:**

Our research demonstrated that most barriers and concerns related to using critical services during a pandemic may be addressed by providing information about available services, providing transportation to facilities, and providing masks to personnel and individuals. CHWs could play critical role in managing and responding to emergencies and pandemics if the government invest on their capacity and motivation. Revision of training curriculum for CHWs and their job description to include the emergency and pandemic management at community level, and providing them monetary incentives are highly recommended.

## Introduction

The COVID-19 pandemic, which emerged in early 2020, has had widespread direct and indirect impacts across all sectors. In particular, low- and middle-income countries (LMICs) have experienced the most significant strain on their health systems. The initial stages of the pandemic witnessed a substantial disruption in health system service delivery, affecting human resources, facility utilization, and medical supplies [1]. Amid this challenge, Community Health Workers (CHWs) emerged as crucial figures, actively engaging in pandemic response like early detection, rapid assessments, and contact tracing [2]. CHWs are pivotal in providing essential health services to their communities, including health promotion, community mobilization, and various preventive and clinical services. As frontline workers, they are often the first point of contact during outbreaks or pandemics [3].

Afghanistan health system consists of two main packages: Basic Package of Health Services (BPHS) and Essential Package of Hospital Services (EPHS). BPHS mainly focuses on primary healthcare including preventive and promotive care and the EPHS focuses on secondary and tertiary healthcare in district, provincial, regional and national hospitals. Afghanistan health system faces multiple challenges such as reduced donor support beyond 2018, sub-optimal health services utilization due to distance to health facilities and poverty, shortage of female healthcare providers and insecurity, inadequate country resource for health and low capacity for management and implementation of health projects especially at sub-national level [4–5]. Afghanistan introduced the Community Health Workers posts in the health services in 2005 following the initiation of the Basic Package of Health Services (BPHS). Most CHWs are illiterate; only few can read and write. Health posts, staffed by one female and one male CHW, cover catchment areas of 1000–1500 people. These health posts provide limited curative care along with health promotion and preventive interventions [6]. According to a 2023 National HRH survey, Afghanistan has 27,547 CHWs, with 45% being females [7]. CHWs in Afghanistan primarily focus on essential health services for communicable diseases such as malaria, diarrhoea, and acute respiratory infections like pneumonia, as well as maternal, child, and reproductive health services. Additionally, they provide growth promotion, nutrition counselling, and micronutrient supplementation [8]. CHWs undergo a standard training program consisting of three 18-day courses with two months of fieldwork between each course [6].

Different studies underscore the significant contribution of CHWs in responding to and controlling the COVID-19 pandemic while striving to deliver essential health services in different countries. In Iran, CHWs took on responsibilities such as household screening by phone, contact tracing, and disseminating warnings about the pandemic through hotlines. They were actively involved in identifying inactive cases within their catchment areas [9]. Similarly, in Pakistan, CHWs played a key role not only in controlling and preventing the pandemic but also in providing essential health services, particularly in maternal and child health [10]. CHWs in India are usually women from the communities they serve. They are the backbone of rural primary health care and provide health promotion and prevention services to the community. During the pandemic, they provided additional services such as promoting preventive behaviours, mobilizing testing, contact tracing, delivering medicines, ensuring home quarantines, and supporting COVID-19 vaccination [11].

CHWs were the first point of contact for the Afghan community to obtain information or receive basic services during the pandemic too [12]. They were a bridge between unofficial and official health system and were a certain link with the community. They have highly effective role in increasing demand for Antenatal care (ANC) and vaccination, provision of contraceptive and management of sick children [13]. Although, the government made little efforts to make the CHWs engaged in the pandemic response [14], there are few success stories of CHWs contribution to the control of COVID-19 pandemic in Afghanistan. Urban community health workers (uCHWs) project was implemented by an international NGO in Afghanistan operating in Kabul and Balkh provinces. These uCHWs were trained on COVID-19 screening, detection, and referral of cases as well as risk communication and community engagement. They also continued provision of GBV and SRH services, and referrals to midwives at community-based health centres run by the same NGO. These home-based health posts run by uCHWs played a critical role in filling gaps when health services were shut down or disrupted in static health facilities. They also built a trust among the community by visibly practicing COVID safety measures [15]. Another example is the CHWs engagement in WASH program implemented by UNICEF in Afghanistan. In WASH response, UNICEF prioritized provision of sanitation, safe drinking water, handwashing facilities and hygiene supplies for returnees and internally displaced persons. In this program, CHWs have distributed soap to 406,000 households in locations which were at high risk for polio mainly in Eastern and Southern regions of Afghanistan [16]. However, it is worth mentioning that similar to other healthcare workers, they experienced many mental distresses during the pandemic [17]. Female CHWs also were exposed to threats, violence and harassment as well as insecurity in their catchment area [18]. Given their frontline role, CHWs served vital roles in providing community health services during the COVID-19 pandemic and they can help to identify existing barriers in seeking and delivering community health services in Afghanistan. The primary objective of this study is to uncover these barriers and understand the challenges faced by the Afghan community in seeking and receiving essential health services. It will help the policy makers and program managers to design the effective policies, interventions and program to maximize the CHWs performance and effectiveness for controlling and responding to the pandemics and emergencies. The research questions in our study are as following:

A: What are the barriers to seeking essential health services in communities before and during the COVID-19 pandemic in Afghanistan?
B: What are the challenges and barriers to the delivery of community-based services by CHWs in Afghan society?

## Methods

We used a cross-sectional study design to interview CHWs in 9 provinces of Afghanistan (Kabul, Nangarhar, Kandahar, Balkh, Herat, Daykundi, Nooristan, Kunduz, and Badghis). We used a two-stage random sampling method to select a representative sample of health facilities. We selected nineprovinces from seven regions of Afghanistan, covering approximately 45% of the total population. An advisory committee selected the provinces. This advisory committee consisted of representatives of different organizations (WHO Afghanistan and directorates of MoPH). The main selection indicator was inclusion of provinces with different status (a mix of big cities and underserved provinces from seven zones of Afghanistan). After randomly selecting 49 districts out of 130 districts in these provinces, we randomly selected 105 primary health care clinics. The data collectors interviewed one hundred and ten CHWs (13 in urban and 97 in rural settings) who were available in their clinics. In this study, we used the standard and validated questionnaire “Community Needs, Perceptions and Demand, Community Assessment Tool” which developed by WHO headquarter. The questionnaire was modified by WHO Afghanistan for the local setting of the Afghanistan health system. Then, it was validated by national experts in a national workshop in January 2022. The data collectors were selected based on previous similar experience on national surveys and other research. They received a three-day training on how to use data collection tool, tips of a good interview and development and management of their data collection plan in provinces. The data were collected during September 2022 simultaneously in all selected provinces and entered electronically using the offline Lime Survey application [19] and uploaded to a secure database. To control the quality, a data manager and a survey supervisor were hired. The survey supervisor monitored the data collection process for not missing any data collection point (health facility) and the data manager did the cross-checking for the accuracy of entered data. The main challenge for data collection were coordination with Provincial Public Health Department (PPHD) and implementing NGOs in each province. To address this challenge, the MoPH issued a letter to PPHDs and introduced the data collectors in each province and explained the duration and purposes of their work. The questionnaire has 161 questions and took 40 min to complete. In this study, we only report the findings of “barriers to seeking essential health services in communities” and “barriers to delivery of community-based services”. The questions in these two sections focused on the main reasons people did not receive the health services before and during the pandemic, the first contact to seek advice or receive care when people feel unwell, what type of training CHWs received, the risks during the COVID-19 pandemic for CHWs and their unmet needs to do their work in the health system. The data was exported into Excel, cleaned by the survey data manager for any inconsistencies, repetition and data entry errors, and then imported into and analyzed using STATA version 17. We calculated the frequency distribution of characteristics and responses to the questions. We provided the frequency for missed data in each table and considered the missing data while analyzed it.

### Ethics approval and consent to participate

The study protocol was reviewed and approved by the Afghanistan MoPH’s institutional review board (IRB code no: A.0122.0389). Verbal informed consent was obtained from each CHW. CHWs also were informed that they are free to leave the interview at any stage and that will not affect their employment status and will not cause them any retaliation measures. To maintain the confidentiality, each participant was allocated a code during the interview to keep them anonymous.

## Results

### Characteristics of CHWs

Most of the 110 community health workers were male (80.9%) (Table 1). Their mean age is 36.6 years (with a standard deviation of 11.7). More than half of CHWs (58.1%) worked as volunteers, and 87.2% were in rural areas.

**Table 1:** Characteristics of community health workers in nine provinces of Afghanistan

Characteristics	Total (*N* = 110)	
	*n*	(%)
Gender(missing = 2)		
Female	19	17.2
Male	89	80.9
Age (mean, SD)		
	36.6	11.7
Occupation		
Community leader (e.g. village elder, chairperson of local board or institution)	3	2.7
Community health care worker (paid)	38	34.5
Community health care worker (volunteer)	64	58.1
Others	4	3.6
Residential area		
Urban	13	11.8
Rural	96	87.2

### Barriers to seeking essential health service before the pandemic

CHWs reported various barriers to accessing healthcare services. For instance, lack of information about existing serves within health facilities and lack of transportation were the main barriers. About 66.3% reported that lack of information about available services was a main barrier (Table 2). Other reported barriers were a lack of transportation to health facilities (47.2%), too far from health facilities from their home (40.9%), and a perceived lack of medicines at facilities (23.6%). During the COVID-19 pandmic, 50.9% of CHWs reported no changes in people’s experiences of accessing healthcare.

**Table 2:** Barriers to seeking essential health services in communities before the COVID-19 pandemic (*N* = 110)

	*n*	(%)
Informational and cultural reasons		
Not knowing about available services	73	66.3
Traditional or folk medicines are preferred	26	23.6
Physical access and cost reasons		
Health facilities too far	45	40.9
Lack of transportation to facilities	52	47.2
Lack of transportation for referral between facilities	30	27.2
Service fees too high	19	17.2
Informal payments or bribes are expected	5	4.5
Facility reasons		
Perceived lack of health workers at facilities	19	17.2
Perceived lack of medicines at facilities	26	23.6
Perceived lack of equipment at facilities	14	12.7
Perceived lack of culturally or religiously sensitive services	17	15.4
Disrespectful providers at facilities	5	4.5
Mistrust of providers or facilities	3	2.7
Discrimination against certain communities	4	3.6
Inconvenient opening hours	2	1.8
Long wait time	24	21.8
Other	9	8.1
Changes in people’s experience in getting health care during the COVID-19 pandemic		
No changes	56	50.9
Moderately changed	38	34.5
Strongly changed	12	10.9

### Barriers to seeking essential health services during the pandemic

As shown in Table 3, CHWs also reported that fear of getting infected with COVID-19 at facilities (32.7%), and fear of getting infected with COVID-19 by leaving the house (30.9%) were the main barriers to seeking healthcare. In addition, CHWs believed that disruption in public transportation (19%) and a drop in household income during the pandemic (18.1%) were the main barriers related to physical access and cost. Among the reasons related to health facilities, 10% of CHWs believed that longer wait times at facilities were the main barrier to seeking health care. CHWs also reported that the first contact point to seek advice or receive care when the community feels unwell was mainly CHWs (87.7%), hospitals (65.4), and dispensaries or health posts (37.2%).

**Table 3:** Barriers to seeking essential health services in communities during the COVID-19 pandemic (*N* = 110)

	*n*	%
Reasons related to information, perception and government recommendations		
Fear of getting infected with COVID-19 at facilities	36	32.7
Fear of getting infected with COVID-19 by leaving house	34	30.9
Recommendations to the public to avoid facility visits for mild illness during the pandemic	19	17.2
Recommendations to the public to delay routine care visits until further notice during the pandemic	9	8.1
Not knowing where to seek care during the pandemic	12	10.9
Reasons related to physical access and cost		
Lockdown, curfew or stay-at-home order	10	9
Disruption in public transportation	21	19
Household income dropped during the pandemic	20	18.1
Lost health insurance during the pandemic	11	10
Higher cost because of unavailability of regular care provider (e.g. need to go to providers who charge higher fees)	7	6.3
Reasons related to health facilities		
Facility closure due to COVID-19	5	4.5
Reduced or changed opening hours at facilities due to COVID-19	5	4.5
Provision of specific services suspended at facilities due to COVID-19	6	5.4
Disrupted or poor service provision at facilities due to COVID-19 (limited availability of medicines, commodities and staff)	9	8.1
Longer wait times at facilities because of the current crisis context	11	10
Other	1	0.9
The first contact point to seek advice or receive care when the community feel unwell		
Community health worker	96	87.7
Dispensary or health post	41	37.2
Hospital	72	65.4
Pharmacist or drug/medicine shop	24	21.8
COVID testing centre	22	20
COVID phone line	6	5.4
Other trained healthcare provider	13	11.8
Traditional healer	14	12.7
Internet or virtual forum	6	5.4
Other	1	0.9

### Barriers to the delivery of community-based health services

As shown in Table 4, more than half of CHWs reported that they received training on how COVID-19 spreads (67.2%), COVID-19 vaccine (65.4%), and how to use a mask properly while working (56.3%). More than half of CHWs also believed that the risk of contracting COVID-19 at their work was less than moderate (73%). However, they reported that the main reasons for contracting COVID-19 during work were contacting many people (40.9%) and not having adequate equipment such as masks (27.2%). And 45.4% of CHWs reported that they sometimes feel stigmatized by people in the community fearing CHWs might transmit COVID-19. In addition, many of the CHWs (43.6%) believed that they received some support they required for performing their job responsibilities. Their demand for support were mainly monetary support (37.2%), personal protection equipment (37.2%), training or information on the COVID-19 vaccine (36.3%), training or information on how to protect themselves from COVID-19 while working (33.6%), and training or information on how to prevent transmission of COVID-19 in the community (33.6%). In conclusion, the provided training and the usage of masks were the main barriers to the delivery of community-based health services among CHWs in the Afghanistan context.

**Table 4:** Barriers to delivery of community-based services by community health workers (*N* = 110)

	*n*	(%)
Received information or training about COVID-19 from your supervisor on the following subjects		
How COVID-19 spreads	74	67.2
How to use a mask properly while working	62	56.3
COVID-19 vaccine	72	65.4
Risk of contracting COVID-19 in work		
No risk	17	15.4
Slight	29	26.3
Moderate	29	26.3
High	16	14.5
Very high	8	7.2
Reasons for contracting COVID-19 during work		
Contacting many people	45	40.9
Not having adequate equipment (such as masks)	30	27.2
Not having been vaccinated for COVID-19	16	14.5
My age or underlying health conditions	7	6.3
Long work hours	17	15.4
Using public transportation to commute or to make home visits	13	11.8
The general public not following the guidelines to prevent the transmission	11	10
Feel stigmatized by people in the community fearing CHWs might transmit COVID-19 (missing = 10)		
Never	41	27.3
Sometimes	50	45.4
Often	9	8.1
Receipt rate of required support to properly perform job responsibilities (missing = 10)		
Receive most support	33	30
Receive some support	48	43.6
Receive little support	19	17.2
Unmet support for CWHs		
Monetary support	41	37.2
Personal protection equipment	41	37.2
Other supplies, commodities and equipment to deliver care	31	28.1
Training or information on how to protect themselves/himself from COVID-19 while working	37	33.6
Training or information on how to prevent transmission of COVID-19 in the community	37	33.6
Training or information on COVID-19 vaccine	40	36.3
Training or information on what to do with people with suspected symptoms of COVID-19	31	28.1
Training or information on other issues related to COVID-19	31	28.1
Training and information related to usual work not related to COVID-19	26	23.6
Support for transport	30	27.2
Health insurance	16	14.5
Other	3	2.7

## Discussion

This study highlights that, according to Community Health Workers (CHWs), the main barriers to accessing healthcare services within the Afghan community were attributed to lack of information about available services, and lack of transportation before the pandemic. However, during the pandemic, the primary obstacles shifted, with the main reasons becoming the fear of contracting COVID-19 in healthcare facilities or when leaving homes, disruptions in public transportation, a decline in household income, and longer wait times at facilities. Furthermore, the key challenges in delivering community-based health services during the pandemic were inadequate personal protection equipment for CHWs, a lack of monetary support, insufficient training on various subjects related to the pandemic, and the stigmatization of CHWs by members of the community.

Based on our study, the majority of CHWs are male working volunteer-based and live in rural areas. Gender barriers for selecting women as CHWs is a challenge in Afghanistan [20] while CHWs in other countries like India or Iran are mostly female [11, 21]. A study in Tanzania shows that male and female CHWs are equally able in terms of knowledge and performance, however, they are unequally accepted by the community. Women tend to disclose reproductive and pregnancy concerns more readily to female CHWs, while men are more comfortable discussing sexual and reproductive concerns with male CHWs [22]. So, to have high acceptability and productivity, it is highly recommended to deploy paired male and female CHWs throughout the community. Although, this has been already considered in national policies, however, it needs to be implemented across the country.

Fear of contracting COVID-19 at healthcare facilities also was a significant barrier to seeking essential health services by the community. This negative attitude will affect patients and their families as well as health care providers. It could also lead to disruption in case identification and surveillance of patients [23]. To address this fear, the government, through the media, should provide enough, updated, and regular information for the public. They can also use reference groups such as religious leaders, sports heroes, and other socially acceptable people to convey health messages. Sharing the success stories of people infected and recovered in different regions also can be a good strategy. A study in central and eastern Europe showed that specific groups such as younger older adults, educated individuals, women, and those with poorer health and more chronic conditions were more likely to avoid going to health facilities during the pandemic [24]. However, these groups can be different in different countries, so we recommend the government of Afghanistan identify those people via different methods and provide them with health care through different approaches, such as home-based care or online consultations.

Another main barrier to seeking essential health services for the community was financial hardship and a drop in household income. Unfortunately, the Afghan community pays 77.2% of their health expenditure from their pocket [25]. Addressing this high amount of out-of-pocket (OOP) payment and filling the gap needs systematic and strategic interventions at the country level. The government should explore context-specific solutions and approaches to decrease this high OOP. The establishment of a health insurance system for public employees, strategic purchasing of health services from the public and private sector by a third party, and the establishment of charity funds could be some appropriate solutions.

CHWs experienced different barriers to deliver community-based services during the pandemic. The most highlighted ones especially in Low- and Middle-income countries are stigma and discrimination, transport restrictions, inadequate PPE and tools, lack of guidelines, high workload, and low motivation and poor remuneration [26, 27] (https://www.researchsquare.com/article/rs-3851192/v1). CHWs in Afghanistan also faced with similar challenges from which the most highlighted ones are inadequate PPEs, insufficient training, poor monetary support and experiencing stigma and discrimination. Providing regular training related to COVID-19 for CHWs is also very essential for controlling and preventing the pandemic [28]. Our study indicates that CHWs received formal on the job training and supervision on specific topics, such as the COVID-19 vaccine, its spread, and mask usage, from their supervisors. However, CHWs expressed that they need additional trainings on self-protection and Infection Prevention Control (IPC). So, protecting the healthcare workers in terms of ensuring a good IPC and provision of Personal Protection Equipment (PPE), continuous training, dedicated supervision, and performance management were highly recommended for CHWs during the pandemic or any outbreak in the country [29]. In addition, the government should provide good monetary incentives to CHWs to make them motivated especially during the pandemic.

Our study had two main limitations. First, to obtain the community insights, we interviewed the community health workers (CHWs) not the individual people from the community. Second, there were not any open-ended questions or any focus group discussion to gain more nuanced information which would be very helpful to understand the implications of the findings. We proposed conducting focus group discussions, especially with community members such as Mula Imams and Health Shuras, directly. In addition, population-based surveys enrolling individual people from the community can address the limitation of our study in future.

Besides these limitations, our study showed that awareness of available services, offering transportation services to facilities, and offering masks to people and staff can address most barriers and concerns of using essential services during a pandemic. The Afghanistan ministry of public health mainly Community Based Health Care department, WHO and UNICEF who are the main policy actioners toward CHWs work in Afghanistan can use the findings of this study to revise the training curriculum of CHWs, their job description to include the management of emergencies and pandemics at community level, and to provide them incentives in future. In addition, program managers of implementing NGOs in each province can use the findings of this study for evaluation of CWHs performance and their need assessment. We recommend designing research on how to address and mitigate the stigma and discriminations during the pandemic toward community health workers in Afghanistan too.

## Conclusion

Our research demonstrated that most barriers and concerns related to using critical services during a pandemic may be addressed by providing information about available services, providing transportation to facilities, and providing masks to personnel and individuals. CHWs could play critical role in managing and responding to emergencies and pandemics if the government invest on their capacity and motivation. Revision of training curriculum for CHWs and their job description to include the emergency and pandemic management at community level, and providing them monetary incentives are highly recommended.

## Data Availability

The datasets generated and analyzed during the current study are not publicly available as the informed consent applied only for the use by the research team. If desired, the data can be viewed and reviewed together with the corresponding author.

## Abbreviations

CHW: Community health workers
LMICs: Low- and middle-income countries
BPHS: Basic Package of Health Services
IPC: Infection Prevention Control
PPE: Personal Protection Equipment
OOP: Out-of-pocket
